# Posterior Dahl: A Minimally Invasive Method for the Treatment of Localized Posterior Tooth Wear

**DOI:** 10.3290/j.jad.b3837959

**Published:** 2023-01-26

**Authors:** Pauline M.J. Hoekstra-van Hout, Jan G.J.H. Schols, Shamir B. Mehta, Niek J.M. Opdam, Tatiana Pereira Cenci, Bas A.C. Loomans

**Affiliations:** a Postgraduate Student, Section Orthodontics and Craniofacial Biology, Department of Dentistry, Radboud University Medical Center, Radboud Institute for Health Sciences Nijmegen, the Netherlands. Conception, data acquisition, data analysis and interpretation, drafted and finalized the manuscript, gave final approval for submission of the manuscript and agreed to be accountable for all aspects of the work.; b Professor, Section Orthodontics and Craniofacial Biology, Department of Dentistry, Radboud University Medical Center, Radboud Institute for Health Sciences Nijmegen, the Netherlands. Conception, design, critically revised the manuscript, gave final approval for submission of the manuscript, agreed to be accountable for all aspects of the work.; c Professor, College of Medicine and Dentistry, Birmingham Campus, Ulster University, UK. Conception, design, critically revised the manuscript, gave final approval for submission of the manuscript, agreed to be accountable for all aspects of the work.; d Associate Professor, Department of Dentistry, Radboud University Medical Center, Radboud Institute for Health Sciences Nijmegen, the Netherlands. Conception, design, data acquisition, data interpretation, critically revised the manuscript, gave final approval for submission of the manuscript, agreed to be accountable for all aspects of the work.; e Assistant Professor, Department of Dentistry, Radboud University Medical Center, Radboud Institute for Health Sciences Nijmegen, the Netherlands. Conception, design, data acquisition, data interpretation, critically revised the manuscript, gave final approval for submission of the manuscript, agreed to be accountable for all aspects of the work.; f Professor, Oral Function and Restorative Dentistry, Department of Dentistry, Radboud University Medical Center, Radboud Institute for Health Sciences Nijmegen, the Netherlands. Conception, design, data acquisition, data interpretation, critically revised the manuscript, gave final approval for submission of the manuscript, agreed to be accountable for all aspects of the work.

**Keywords:** restorative dentistry, direct resin composite restorations, Dahl concept, vertical dimension of occlusion, bite raising, tooth wear.

## Abstract

**Purpose:**

This retrospective case series of 9 patients aimed to describe clinical outcomes and patient satisfaction following the implementation of the posterior Dahl concept to manage localized posterior tooth wear.

**Materials and Methods:**

Localized occlusal space was created in the posterior dentition. Supra-occluding direct restorations were placed bilaterally for the restoration of molars. Intraoral scans were taken at the pre-treatment stage, immediately post-restoration, and during follow-up appointments. Scans were used to undertake analysis of any occlusal changes and re-establishment of the occlusion. A questionnaire was used to assess patient satisfaction, alleviation of any pre-treatment concerns, and evaluation of post-treatment complaints.

**Results:**

Immediately post-treatment, all patients showed an increase in the vertical dimension. Opening of the bite in the untreated areas following restoration of worn posterior molars resulted either in a tendency towards or the actual re-establishment of the occlusion. One patient completely lacked compensatory vertical tooth movement in the untreated areas, culminating in the persistence of a vertical open bite. One restoration displayed cohesive fracture after 4 months. Pre-treatment problems (eg, sensitivity) were fully resolved amongst all patients after 6 months. Post-treatment complaints were minor and demonstrated resolution within a relatively short period of time. Eight patients reported being “very satisfied” with their treatment outcomes.

**Conclusion:**

Application of the posterior Dahl concept appears to offer a promising, relatively simple, minimally invasive and effective approach for the management of localized posterior tooth wear, which is well accepted by patients.

In restorative dentistry, the clinical management of anterior localized tooth wear by application of relative axial tooth movement, commonly referred to as the Dahl concept, is a well-known treatment modality.^10,15^ The concept was initially introduced in 1975 and is based on the planned increase in the vertical facial height by means of a partial bite-raising splint in the incisor and canine area for patients with localized, severe anterior tooth wear.^3,6^ The placement of the partial bite-raising splints resulted in the disocclusion of the unworn (pre)molars. Over the course of time, full occlusion and intercuspation were regained in the (pre)molar areas by passive eruption of the posterior teeth and intrusion of the anterior teeth. The anterior intra-occlusal space created by the placement of the partial bite-raising splint facilitated restoration of tooth surface loss in the incisor and canine areas without the need for further significant subtraction of tooth tissue from the occluding surfaces.^3,15^

The clinical effects of the Dahl concept are also known as relative axial tooth movement.^8,15^ In 1982 by means of an x-ray cephalometric study, Dahl and Krogstad showed that true axial tooth movement occurred rather than proclination of the anterior teeth.^4,5^ The success rate of re-establishment of occlusion in the posterior dentition following the application of an anterior Dahl appliance has been reported to vary from 83%^9^ to 100%.^4,18^ As part of the re-establishment of occlusion, Dahl et al^4^ suggested that anterior intrusion movements accounted for approximately 40% of the observed axial tooth movement, and posterior eruption for 60% of axial tooth movement. However, a recent review by Goldstein et al^7^ alluded to the lack of compelling evidence to support concepts relating to the direction of tooth movements that are typically observed to take place with the application of the Dahl concept. The time needed for the re-establishment of occlusion has been documented to range from 4.6 months^11^ to 9 months,^9^ and on average takes about 6 months.^8,18^ Relapse was reported by Dahl and Krogstad^6^ within the first six months after discontinuation of splint wear, but was observed to cease thereafter.

The application of a transposed procedure, hence, a “posterior Dahl concept” with interocclusal space creation in the anterior area as opposed to the posterior area, may be expected to lead to comparable clinical effects (though inverted with respect to the posterior and anterior teeth). Banerji et al^1^ described the application of unilateral supra-occluding restorations for the minimally invasive management of Cracked Tooth Syndrome (CTS). Chana et al^2^ described the bilateral placement of posterior restorations in a supra-occlusal vertical dimension. The primary goals of both of the latter studies were to evaluate the efficacy of the supra-coronal splinting of cracked teeth as well as restoration survival. Both studies concluded that supra-occlusal restorations placed in the (pre)molar area can offer a successful and minimally-invasive treatment modality for the management of CTS, for instance.

Based on the same clinical principles, a posterior Dahl concept may also be applied for the management of localized posterior tooth wear (in the absence of anterior tooth wear). Dentists have limited or no minimally invasive technical treatment options for managing localized posterior tooth wear in symptomatic patients. Conventional treatment in these cases typically consists of full-mouth bite elevation on all teeth, involving extensive restorative work. Other options under such circumstances include orthodontic intrusion or further mechanical reduction of the occlusal surfaces to create intra-occlusal space to accommodate restorative materials, followed by the restoration of the affected teeth. Patients with localized posterior tooth wear may therefore benefit from the application of the minimally invasive posterior Dahl concept.

Recently, a paper by Tew et al^19^ described two case reports in which localized posterior tooth wear was treated using the posterior Dahl concept. However, as far as the current authors know, the application of the posterior Dahl concept has not been examined in larger case series or retrospective studies. The aim of the present retrospective case series is to describe the clinical outcomes (re-establishment of occlusion, mandibular midline deviation, occlusal changes, and restoration failure) as well as the levels of patient satisfaction (pain, chewing, biting, and speech problems) following the application of the posterior Dahl concept for the management of localized posterior tooth wear in a sample of nine patients where complete documentation was available.

## Materials and Methods

### General

This study was conducted in full accordance with regulations by the World Medical Association Declaration of Helsinki, and ICH E6 (R2) Guidelines for Good Clinical Practice (GCP). Ethical approval was obtained from local Human Research Ethics Committee (case number 2022-15767).

### Patient Selection

Patients were treated with the posterior Dahl concept, either at the Department of Dentistry of Radboud UMC in Nijmegen, or at the private dental practice of one of the authors (BL). The etiology of tooth wear in these patients was either mechanical and/or chemical (Table 1). All patients who were selected needed treatment due to symptoms of sensitivity, threatening endodontic complications, or functional problems. The participants’ Tooth Wear Index (TWI) scores varied from 2 to 4. However, the primary reason for restoration was not the amount of tooth wear, but the reporting of pain and/or functional problems.

**Table 1:** Overview of patient characteristics, intra-oral scan recalls and treatment details

Patient #	Sex (M/F)	Age at treatment (y-m)	Estimated etiology of wear	TWI pre-treatment	1st recall scan (months)	2nd recall scan (months)	3rd recall scan (months)	4th recall scan (months)	Teeth and materials used
1	F	28-9	Multi-factorial	2	3	7	–	–	37 AP-X 47 AP-X
2	F	33-5	Mechanical (bruxism)	2	2	4	8	20	37 GiC 47 AP-X
3	M	44-9	Multi-factorial	3	1.5	6.5	–	–	36 GiC 46 AP-X
4	M	18-6	Multi-factorial	2	3.5	–	–	–	16 AP-X 26 AP-X
5	M	20-2	Chemical (acids)	3	4.5	12	49	–	36 AP-X 46 AP-X
6	M	43-7	Multi-factorial	3	3	–	–	–	36 AP-X 46 AP-X
7	M	47-5	Mechanical (bruxism)	4	4	–	–	–	37 AP-X 47 AP-X
8	M	70-5	Mechanical (bruxism)	2	9	18	36	–	17/16 AP-X 27/26 AP-X
9	M	46-2	Multi-factorial	3	1.5	8	–	–	37 AP-X 47 AP-X

GIC: glass-ionomer cement; AP-X: Clearfil AP-X.

Treatments were performed between October 2014 and April 2021. Retrospectively, the authors were able to retrieve complete intraoral scan-sets for nine patients. Intraoral scans were taken at the pre-treatment stage, immediately post-treatment and at successive follow-up appointments. Written informed consent to participate was obtained from all patients, as well as agreement for the use of anonymized intraoral scan data.

### Treatment: The Posterior Dahl Concept

All patients were treated by one of the authors (BL or NO), both following the same clinical treatment protocol and both using the same materials. In the nine patients, the effects of localized posterior tooth wear – culminating in either functional-, sensitivity- or pain-related problems – were managed by the application of supra-occlusal, directly bonded resin composite restorations. The post-treatment vertical dimension of occlusion (VDO) was estimated by the amount of resin composite required for restoration of the anatomical proportions of the affected teeth.

A minimally invasive procedure was applied, including minor roughening of the occlusal surfaces of the affected teeth using a diamond bur. In the event of an existing but insufficient restoration, this restoration was either replaced or repaired. Isolation of the operating field was achieved using rubber-dam, or the use of cotton rolls and suction devices. If required, appropriate matrix systems and wedges were also used. A 3-step etch-and-rinse adhesive was applied in accordance with the respective manufacturer’s instructions: 37% phosphoric acid (DMG; Hamburg, Germany), Clearfil SA Primer, and Clearfil Photobond (Kuraray Noritake; Tokyo, Japan). A microhybrid composite material (Clearfil AP-X, Kuraray Noritake) was used to restore the anatomical form of the treated teeth (approximately 1.0 – 1.5 mm supra-occlusal thickness). An LED polymerization unit with a minimal output of 1000 mW/cm^2^ was used to light cure the restorations. Minimal occlusal morphology was applied in the restorations, and occlusal contacts were positioned in the center of the restoration(s).

In the case of a unilaterally worn tooth, glass-ionomer cement (GIC, Fuji TRIAGE, GC; Tokyo, Japan) was placed on the contralateral molar, which was also elevated to the same occlusal plane as the definitively restored worn tooth. This was done to provide the patient with both bilateral occlusal stops on molars and also improved comfort during the procedure. All other teeth were left out of occlusion directly after treatment.

### Intraoral Scanning and Recalls

Intraoral 3D scans were taken immediately pre- and post-treatment and at all subsequent recall visits. Recall visits differed in terms of the number of months after treatment (Table 1). Scans were made using either a True Definition Scanner (3M Oral Care; St Paul, MN, USA) or a Medit i500 scanner (Medit; Seoul, South Korea), processed with open-source software MeshLab v2020.12 (http://www.meshlab.net). Patients sat in an upright position during scanning, with an OptraGate lip and cheek retractor inserted (Ivoclar Vivadent; Schaan, Liechtenstein).

### Qualitative Analysis

All intraoral scans were analyzed by one author (PH). All pre- and post-treatment scans were scored for:

- Eruption of untreated (pre)molars (yes/no)
- Eruption from canine to canine (yes/no)
- Midline deviation (in mm)
- Angle classification of the first molars
- Any other remarks

### Questionnaire

All patients received a short retrospective questionnaire to assess symptoms of discomfort, pain, functional problems, and their experience before and after treatment. The questionnaire items included:

- Presence of pain in the TMJs, musculature or teeth
- Presence of functional problems regarding frontal biting of food, posterior chewing of food, or speech
- Presence of discomfort regarding cheek biting or failure of restorations
- Did the treatment resolve the pre-treatment concerns?
- Overall (dis)satisfaction with this treatment method
- General remarks from patients

All items were evaluated retrospectively (ie, after treatment and follow-up, merely for this study) by telephone interviews by one of the authors (PH) at the following timepoints: pre-treatment, immediately post-treatment, 1 week post-treatment and 2 months post-treatment. Except for the expression of “overall (dis)satisfaction” (scored using a 5-point verbal rating scale from very dissatisfied to very satisfied), all other answers were scored using a 3-point verbal rating scale (yes, no, or I can’t remember).

## Results

### Patients and Treatments

An overview of patient characteristics, intraoral scan recalls, and treatment details are presented in Table 1. Although no adverse events occurred during treatment, two restorations in one patient (number 7) had to be replaced after 4 months. This was due to partial fracture and wear (see “Questionnaire” below and Fig 3). Seven patients were treated bilaterally with Clearfil AP-X composite. Two patients presented with unilateral posterior localized tooth wear; the affected ipsilateral molars were restored using Clearfil AP-X composite, and GIC glass-ionomer cement was applied on the contralateral tooth where definitive restorative rehabilitation with resin composite was not indicated. The maximum follow-up period varied from 3 to 49 months, with a mean of 15 months (Table 1).

### Qualitative Analysis

**Fig 1:**
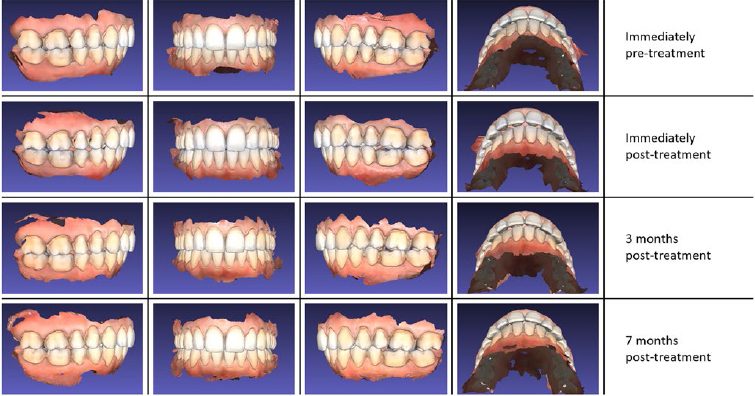
Overview of intra-oral scan images of patient 1.

Patient 1 is presented as a representative example in Fig 1. Qualitative results of all patients are presented in Table 2, in which the clinical situation at the latest recall visit is given.

**Table 2:** Qualitative analysis of posterior Dahl concept treatment effects

Patient #	(Pre)molar eruption Bite closing tendency	Frontal eruption Bite closing tendency	Mandibular midline deviation	Occlusion change L Pre-treatment – post-treatment	Occlusion change R Pre-treatment – post-treatment	Other remarks
1	+	+	1 mm R	¼ disto – neutro	⅓ disto – ½ disto	Fixed mandibular orthodontic retainer present
2	+	+	<1 mm L	-	-	Frontal open bite did not fully return to baseline
3	+	+	-	-	-	Wear of 11 and 21, restored for esthetic reasons
4	+	+	<1 mm L	-	-	Fixed mandibular orthodontic retainer present
5	–	–	1 mm L	–	–	Long-face/vertical growth pattern with mentalis strain
6	+	+	1 mm R	–	–	–
7	+	+	–	–	–	–
8	+	+	–	–	–	Wear of 23, restored for esthetic reasons
9	+	+	–	–	–	–

All variables were evaluated post–treatment at the latest recall intra–oral scan. L: left; R: right.

In general, all patients showed an increase in the vertical dimension immediately after treatment, leading to slight distalization of the molar occlusion, and an open bite in the anterior area and the non-treated (pre)molars. In seven patients, these effects returned to the baseline situation during the observation period (ie, re-establishment of occlusion and closing of the open bite). Eight patients showed the same pattern of occlusal re-establishment, which appeared to progress from distal to mesial. One patient showed no closing of the open bite over time, either in the frontal area or in the non-treated (pre)molar areas (patient number 5). As a result, this patient was additionally provided with composite restorations on the non-treated (pre)molars 1.5 years after initial treatment. The open bite in the frontal area was left untreated, and did not close over time up until the last recall (49 months after initial treatment). As the patient did not have any complaints, it was decided to leave the open bite unchanged. In one other patient (number 2), the frontal open bite partially closed but did not fully return to the baseline situation.

**Fig 2:**
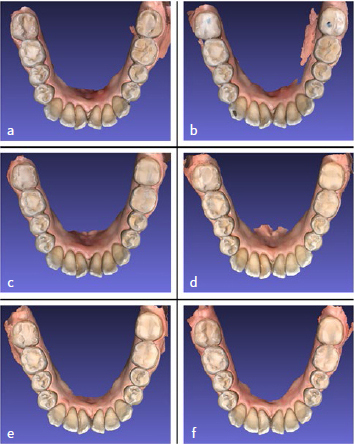
Overview of patient 2 (left and center). Gradual wear over time of the glass-ionomer restoration on tooth number 47 is clearly visible. a: pre-treatment; b: immediately post-treatment; c: 2 months post-treatment, d: 4 months post-treatment, e: 8 months post-treatment, f: 20 months post-treatment.

Two patients received contralateral GIC restorations. These GIC restorations showed progressive wear over time, with the subsequent re-establishment of occlusion of these molars (Fig 2).

In five patients, mandibular midline deviation gradually occurred over time, with a maximum deviation of approximately 1 mm (Fig 1).

### Questionnaire

Table 3 shows the results of the pre-treatment questionnaire. Symptoms of pain in worn teeth were the primary concern for four patients, whereas restoration fracture was reported by two patients.

**Table 3:** Pre-treatment questionnaire evaluation

Patient #	Pain in TMJ	Pain in musculature	Pain in worn teeth	Restoration fracture	GERD
1	–	–	–	–	–
2	–	–	+	–	–
3	–	–	–	–	–
4	–	–	+	–	–
5	–	–	–	–	–
6	+	+	+	+	+
7	–	–	–	–	–
8	–	–	–	+	–
9	–	–	+	–	–

(-): absent; (+): present. GERD: gastroesophageal reflux disease.

The outcomes of the post-treatment questionnaire are given in Table 4. As for pain, the patient with pre-treatment TMJ complaints and chewing musculature pain indicated that these problems had resolved at 2 months post-treatment. Two out of the four patients who complained about pre-treatment pain in worn teeth mentioned resolution of the pain immediately post-treatment. In the other two patients with pain due to tooth wear, the symptoms of pain disappeared within one week or 2 months post-treatment, respectively.

**Table 4:** Post-treatment questionnaire evaluation

Patient #	Immediately post-treatment									One week post-treatment									Two months post-treatment								
	Pain in TMJ	Pain in musculature	Pain in treated (pre)molars	Pain in non-treated teeth	Difficulty with chewing	Difficulty with frontal biting	Difficulty with speech	Cheek biting	Failure of restorations	Pain in TMJ	Pain in musculature	Pain in treated (pre)molars	Pain in non-treated teeth	Difficulty with chewing	Difficulty with frontal biting	Difficulty with speech	Cheek biting	Failure of restorations	Pain in TMJ	Pain in musculature	Pain in treated (pre)molars	Pain in non-treated teeth	Difficulty with chewing	Difficulty with frontal biting	Difficulty with speech	Cheek biting	Failure of restorations
1	–	–	–	–	+	+	–	–	–	–	–	–	–	+	+	–	–	–	–	–	–	–	–	–	–	–	–
2	–	–	–	–	+	–	–	–	–	–	–	–	–	–	–	–	–	–	–	–	–	–	–	–	–	–	–
3	–	–	–	–	+	–	–	–	–	–	–	–	–	–	–	–	–	–	–	–	–	–	–	–	–	–	–
4	–	–	–	–	–	–	–	–	–	–	–	–	–	–	–	–	–	–	–	–	–	–	–	–	–	–	–
5	–	–	–	–	–	–	–	–	–	–	–	–	–	–	–	–	–	–	–	–	–	–	–	–	–	–	–
6	+	+	+	–	+	–	+	+	–	+	+	–	–	–	–	–	–	–	–	–	–	–	–	–	–	–	–
7	–	–	–	–	+	+	+	–	–	–	–	–	–	+	+	+	–	–	–	–	–	–	–	–	–	–	–
8	–	–	–	–	–	–	–	+	–	–	–	–	–	–	–	–	–	–	–	–	–	–	–	–	–	–	–
9	–	–	+	–	+	+	–	–	–	–	–	+	–	+	+	–	–	–	–	–	–	–	+	+	–	–	–

(-): absent; (+): present.

Three patients reported difficulty chewing at the one-day post-treatment evaluation; however, these symptoms were no longer present at the 1-week post-treatment recall. Two other patients described difficulty both with chewing and frontal biting as persisting up to one-week post-treatment. In one other patient, challenges with chewing and biting continued for approximately 6 months.

Other functional problems, such as cheek biting and problems with speech, resolved at one-week post-treatment, except for one patient, in whom speech problems were still present one-week post-treatment.

**Fig 3:**
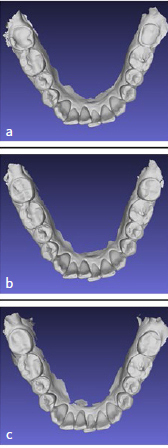
Overview of patient 7 (right), showing fracture (tooth number 37) and wear (tooth number 47) of the composite restorations. a: pre-treatment; b: immediately post-treatment, c: 4 months post-treatment.

Although no patients mentioned failure of restorations of the treated molars within two months post-treatment, in one patient (number 7), two restored molars had to be re-treated due to a partial fracture of one restoration (tooth 37) and wear of the other restoration (tooth 47). The latter took place 4 months post-treatment (Fig 3).

All except patient 7 mentioned that their pre-treatment problems were eventually resolved and gave patient satisfaction the score of “very satisfied”. Patient 7 answered “neutral” on patient satisfaction.

## Discussion

For the restorative management of localized posterior tooth wear, limited treatment options are available. Occlusal reduction and subsequent restoration is perhaps the most obvious treatment method; however, this approach is rather invasive. Occlusal restoration without prior occlusal reduction as described in this study is quite unconventional. This treatment is a largely minimally invasive approach, and treatment with this experimental procedure by one of the authors (inspired by the work of Banerji et al^1^) showed good clinical results. The primary aims of this investigation were to describe the clinical outcomes (re-establishment of occlusion, mandibular midline deviation, occlusal changes, and restoration failure) and the levels of patient satisfaction (pain, chewing-, biting- and speech-related problems) following application of the posterior Dahl concept to manage localized posterior tooth wear in a sample of nine fully documented patients.

A few studies have mentioned using the posterior Dahl concept, but their primary purpose was not to investigate this concept per se. Banerji et al^1^ and Chana et al^2^ used the posterior Dahl concept as a tool rather than the primary subject of their investigations. Recently, a paper with two case reports by Tew et al^19^ presented treatment of localized posterior tooth wear with the posterior Dahl concept to actually describe the concept itself.^19^ However, neither follow-up by means of intraoral scanning nor cast comparison were performed, nor were patients asked about their satisfaction with this approach. With these two elements included, our study adds value to research on the posterior Dahl concept.

The results of our study are comparable to the most relevant study performed on this subject (Banerji et al),^1^ despite the fact that that they applied unilateral supra-occluding restorations instead of bilateral restorations. The main findings by Banerji et al^1^ were complete re-establishment of the occlusal contacts in the anterior teeth in 97.7% (n=128) of their cases at 3 months post-treatment. An overall success rate of the supra-occlusal restorations of 86.7% was reported after 3 months,^1^ including relief of the symptoms associated with CTS. This extra parameter for the definition of success clearly has a detrimental effect on the success rate. The restorations were reported to be well tolerated, with an overall acceptability of 97%.^1,16^

With the conventional application of the anterior Dahl concept, the success rate for the re-establishment of occlusion on posterior teeth after placing supra-occluding anterior restorations to this end has been reported to range from 94% to 100%.^4,8,18^ Furthermore, survival of supra-occluding restorations has also been reported to be as high as 94%.^16^ The results of the studies involving the anterior Dahl concept are comparable with the results of the current investigation applying the posterior Dahl concept, in which only one patient did not display re-establishment of full occlusion on the non-treated teeth, and one restoration showed signs of partial fracture after 4 months.

Failure to close open bite, as reported in the present study amongst two patients, warrants further discussion. One of these patients (no. 5) showed no closure of the open bite over time, either in the anterior area or in the non-treated (pre)molar areas. The vertical facial configuration of this patient was a long-face type, which is known to have a deviating occlusal force pattern compared to individuals with normal vertical facial dimensions.^17^ Since the re-establishment of occlusion is caused by relative and true axial tooth movement,^4,5,8,16^ in which both intrusion of treated teeth and extrusion of non-treated teeth are involved,^4^ the intrusive component in this patient was probably not sufficient to achieve full occlusion in the non-treated areas. For the other patient (no. 2), the frontal open bite displayed partial closure; however, it did not fully return to the baseline situation.

One patient showed fracture of one restoration after 4 months (Fig 3). This patient suffered from severe bruxism and showed multiple restoration fractures even before treatment with the posterior Dahl concept. Occlusal stress is a known risk factor for restoration failure by (chip) fracture,^13,20^ and it may account for the restoration fracture observed in this patient. Van de Sande et al^20^ reported a 4-fold increased risk for restoration fracture amongst patients with bruxism compared to non-bruxing patients. The posterior teeth in this patient were severely worn and no space was available to place a restoration without endodontic treatment. Anticipating that the risk of failure would be higher compared to a “normal” situation, it was collectively decided with the patient to place a supra-occlusal restoration. As the goal of the posterior Dahl treatment in this case was to protect the underlying tooth material, it may be stated that the treatment goals were successfully met.

In the case of a unilaterally worn tooth, a GIC restoration was placed on the contralateral non-worn molar at the same elevated level of occlusion. This was done in two cases to provide these patients with more comfort by delivering bilateral occlusal stops. The rationale behind the use of GIC is the higher wear rate compared to resin composite. The authors expected the GIC restorations to wear faster over time, causing compensatory eruption of the tooth, with eventual re-establishment of occlusion of the non-worn pre-treatment occlusal surface, and full disappearance of the GIC. These GIC restorations did indeed showed progressive wear over time, with the subsequent re-establishment of full occlusion of the pre-treatment occlusal surface of these molars.

Patient satisfaction in this study was reported to be high (the maximum score “very satisfied” was given 8 times, and on one occasion, the middle score, “neutral” was documented). Only one study in the current literature could be found in which subjective patient satisfaction was evaluated after the application of the Dahl concept; however, it used the anterior Dahl method.^12^ In that case series, 5 of 6 patients reported no discomfort after treatment and 1 patient mentioned mild discomfort.

Observational descriptive studies such as this case series are classified as providing a relatively low level of evidence. Nevertheless, they have great value in experimental treatment concepts, to generate hypotheses which can be tested more extensively with other types of studies. In future studies about the posterior Dahl concept, it would be worthwhile to compare active orthodontic bite-closure treatments with respect to treatment time, costs, and patient satisfaction. Furthermore, a prospective trial would make it possible to assess patient-reported outcome measures (PROMs), such as pain and other discomfort, with the use of a quantifiable visual analogue scale (VAS). The latter was not reliable enough in our study due to its retrospective nature. Also, the influence of patient-related factors such as gender, caries risk and activity, oral hygiene, facial type, and bruxism should be studied in the future, as these aspects are known to influence restoration survival.^21^

## Conclusion

Application of the posterior Dahl concept appears to offer a promising minimally invasive and relatively simple yet effective approach for the treatment of localized posterior tooth wear. Although phenomena such as cheek biting, small fractures and midline deviation occurred, they were minor compared to the overall benefit for these patients, who gave “highly satisfied” scores. Re-establishment of occlusion was achieved in almost all cases and is comparable to the anterior Dahl treatment. A prospective trial on this treatment concept is needed to substantiate this method.

## Clinical relevance

The posterior Dahl concept is a minimally invasive technique for the treatment of tooth wear. This adds a relatively simple yet effective method to the clinical armamentarium for managing localized posterior tooth wear.
